# Household Food Insecurity and Cognition in Youth and Young Adults with Youth-Onset Diabetes

**DOI:** 10.1155/2023/6382663

**Published:** 2023-09-14

**Authors:** Andrea D. Brown, Angela D. Liese, Allison L. B. Shapiro, Edward A. Frongillo, Greta Wilkening, Julius Fridriksson, Anwar T. Merchant, Leora Henkin, Elizabeth T. Jensen, Beth A. Reboussin, Amy S. Shah, Santica Marcovina, Lawrence M. Dolan, Dana Dabelea, Catherine Pihoker, Jason A. Mendoza

**Affiliations:** ^1^Department of Epidemiology and Biostatistics, Arnold School of Public Health, University of South Carolina, 915 Greene Street, Columbia, SC 29208, USA; ^2^Department of Pediatrics, School of Medicine, University of Colorado Anschutz Medical Campus, 13123 E 16th Avenue, Aurora, CO 80045, USA; ^3^Department of Health Promotion, Education, and Behavior, University of South Carolina, 915 Greene Street Columbia, Columbia, SC 29208, USA; ^4^Department of Communication Sciences & Disorders, University of South Carolina, 1705 College Street Columbia, Columbia, SC 29208, USA; ^5^Department of Biostatistics and Data Science, Wake Forest School of Medicine, 475 Vine Street, Winston-Salem, NC 27101, USA; ^6^Department of Epidemiology and Prevention, Wake Forest School of Medicine, 475 Vine Street, Winston-Salem, NC 27101, USA; ^7^Department of Pediatrics, Division of Endocrinology, Cincinnati Children's Hospital Medical Center, The University of Cincinnati, 3333 Burnet Avenue, MLC 4002, Cincinnati, OH 45229, USA; ^8^Medpace Reference Laboratories, 5365 Medpace Way, Cincinnati, OH 45227, USA; ^9^Department of Epidemiology, Colorado School of Public Health, Anschutz Medical Campus, 13001 E 17th Place, Mail Stop B119, Aurora, CO 80045, USA; ^10^Department of Pediatrics, University of Washington, P.O. Box 356320, Seattle, WA 98115-8160, USA; ^11^Seattle Children's Research Institute, P.O. Box 5371, Seattle, WA 98145-5005, USA

## Abstract

**Objective:**

We evaluated the association of household food insecurity (FI) with cognition in youth and young adults with type 1 diabetes (T1D) or type 2 diabetes (T2D).

**Design:**

In this cross-sectional study, age-adjusted scores for composite fluid cognition, and sub-domain scores for receptive language and inhibitory control and attention, were stratified by diabetes type using linear regression, with FI in the past year as the predictor, controlling for covariates. Tests for processing speed, inhibitory control/attention, working memory, episodic memory, and cognitive flexibility were administered to measure the composite fluid cognition score. The NIHT-CB Picture Vocabulary Test was used to assess the crystallized cognition score, and rapid identification of congruent versus noncongruent items was used to assess inhibitory control and attention score. *Setting*. The SEARCH for Diabetes in Youth study is representative of five U.S. states. *Participants*. Included 1,574 youth and young adults with T1D or T2D, mean age of 21 years, mean diabetes duration of 11 years, 51% were non-Hispanic white, and 47% had higher HbA1c levels (>9% HbA1c).

**Results:**

Approximately 18% of the 1,240 participants with T1D and 31% of the 334 with T2D experienced FI. The food-insecure group with T1D had a lower composite fluid cognition score (*β* = −2.5, 95% confidence interval (CI) = −4.8, −0.1) and a lower crystallized cognition score (*β* = −3.4, CI = −5.6, −1.3) than food-secure peers. Findings were attenuated to non-significance after adjustment for demographics. Among T2D participants, no associations were observed. In participants with T1D, effect modification by glycemic levels was found in the association between FI and composite fluid cognition score but adjustment for socioeconomic characteristics attenuated the interaction (*p*=0.0531).

**Conclusions:**

Food-insecure youth and young adults with T1D or T2D did not have different cognition compared to those who were food-secure after adjustment for confounders. Longitudinal research is needed to further understand relations amongst these factors.

## 1. Introduction

Social determinants of health, including household food insecurity, are increasingly recognized as root causes of poor diabetes self-management and higher HbA1c levels, and likely contribute to inequities in outcomes for underrepresented racial and ethnic populations with diabetes [1–4]. Household food insecurity is defined as “limited or uncertain availability of nutritionally adequate and safe foods…” [5] and is not only a nutritional hardship but also exerts severe negative influences on mental and physical health [6]. In people with diabetes, the detrimental consequences can include higher HbA1c levels [1, 2], hypoglycemia, and a much higher frequency of hospitalization [1–3, 7, 8].

Research in people without diabetes also suggests that prolonged experiences of food insecurity affect cognition, with the timing of exposure to food insecurity potentially impacting different cognitive domains. For example, food insecurity experienced in early or later life is associated with global deficits in cognitive function, whereas food insecurity in later life is associated with poorer performance in specific cognitive domains, including executive functions and memory [9]. Food insecurity has been found to specifically influence increased persistent hyperactivity and inattention among youth over time [10] and into adulthood [11]. These effects of food insecurity on cognition could be further exacerbated by poor self-management of chronic diseases like diabetes.

A recent SEARCH for Diabetes in Youth (SEARCH) study found that youth with Type 2 diabetes mellitus (T2D) have lower cognition than those with Type 1 diabetes mellitus (T1D), but did not investigate the role of food insecurity on cognition among youth with diabetes [12]. In people with diabetes, higher HbA1c levels affect brain health, including reduced performance in processing information, memory, and executive function and a higher risk of dementia with advancing age [9]. Children with type 1 diabetes (T1D) who experience frequent or severe glycemic extremes have more pronounced cognitive deficits than those with blood sugar levels that are within the target range [13, 14]. Thus, in adults and children with diabetes, higher HbA1c levels may compound the effects of other factors—including food insecurity—on cognitive functioning. To the best of our knowledge, however, no research has focused on evaluating the interrelationship between household food insecurity and cognitive functioning in persons with youth-onset T1D or type 2 diabetes (T2D), despite the role of glycemia as a potential effect modifier. This lack of understanding is problematic, as we have recently shown that more than 17% of youth and young adults with youth-onset T1D and more than 30% of those with T2D experience food insecurity, a prevalence that significantly exceeds estimates for the general US population [15]. Moreover, food insecurity in youth and young adults with diabetes is associated with substantially higher odds of higher HbA1c levels [16–18].

The purpose of the present study was to characterize the cross-sectional association of food insecurity with performance on cognitive tests for composite fluid cognition, crystallized cognition, and inhibitory control and attention, as measured by the National Institutes of Health Toolbox-Cognitive Battery, in youth and young adults with T1D and T2D [19]. We hypothesized that participants experiencing food insecurity would have lower scores on crystallized cognition and fluid cognition, and particularly on the measure of inhibitory control and attention, independent of other socioeconomic and clinical characteristics. We further hypothesized that the negative impact of food insecurity on cognition would be significantly more pronounced among youth and young adults with higher HbA1c levels.

## 2. Materials and Methods

### 2.1. Study Subjects

SEARCH was initiated in 2001 and is a multi-center observational cohort study of physician-diagnosed diabetes mellitus in youth diagnosed before the age of 20 [20, 21]. Data collection sites include South Carolina, Ohio, Colorado, Washington, and California. SEARCH was initially designed as a multi-center surveillance effort aiming to assess the incidence and prevalence of youth-onset diabetes and was expanded to a cohort study with the start of SEARCH 3 (2010–2015). The inclusion criteria for the SEARCH 3 cohort included (1) a diagnosis date in 2002–2005, 2006, or 2008, (2) having completed a baseline in-person visit, and (3) having at least 5 years of diabetes duration at the time of the visit. During SEARCH 4 (2015–2020), the cohort was operationally split into the group invited to yet another study visit and those who were only invited to complete surveys (survey-only). Those invited to the in-person visit included all those with T2D, all minority racial and ethnic groups, and a random sample of non-Hispanic whites with T1D who were all 10 years or older. In addition, the SEARCH 4 study visit group was supplemented by SEARCH participants who were first diagnosed in 2012 and completed a registry in-person visit during SEARCH 3.

The SEARCH study was approved by and followed procedures in accordance with the ethical standards of the respective local institutional review boards. Before data collection commenced, parents of participants under the age of 18 provided written informed consent, while minor participants over the age of 8 provided assent; all participants aged 18 years or older provided written informed consent.

This study uses data from participants who completed in-person assessments during SEARCH 4 cohort study (*N* = 1,574), where cognitive testing was performed. SEARCH 4 participants were on average 20.8 years old (range 10.1–36.0) with an average of 11.2 (range 4.9–17.4) years of diabetes duration at the time of cognitive testing. Data on food security status and cognition were not available prior to SEARCH 4.

### 2.2. Study Measures

#### 2.2.1. Household Food Security Status

Household food insecurity was assessed using the 18-item United States Department of Agriculture's (USDA) Household Food Security Survey Module (HFSSM), which queries the previous 12 months [5]. The HFSSM was completed by parents/guardians of participants who were minors and by participants ≥18 years of age. The first 10 questions pertain to all households (with or without children), and the last 8 questions are only asked of households with children ages 0–17. Both households with and without children were classified as being food insecure if three or more food insecure conditions or behaviors were affirmed, whereas two or fewer affirmations were categorized as being food secure [5].

#### 2.2.2. Cognition Measures

SEARCH used the National Institutes of Health Toolbox (NIH Toolbox) [22] section on cognitive function (NIHT-CB) which includes a set of brief, psychometrically sound measures currently administered via an iPad and which have been validated in participants ages 3–85 years. Five separate tests were administered, allowing for rapid measurement of a composite score for fluid cognition that included sub-domain scores for processing speed, inhibitory control/attention, working memory, episodic memory, and cognitive flexibility. The measures required distinguishing matching from non-matching pictures over a 90 s interval (Processing Speed), rapid identification of congruent versus noncongruent items (Flanker Inhibitory Control and Attention), recall of sets of items presented visually and auditorily ordered by size and category (List-Sorting Working Memory), matching by named categories with inhibition of response to non-named categories (Dimensional Card Sorting Test), and recall of a series of pictures presented in non-anticipatable order (Picture Story Memory). The NIHT-CB Picture Vocabulary Test was used to assess one area of crystallized cognition, or acquired knowledge, specifically receptive language. This measure required that the examinee identify the picture that matched the aurally named item. Both phonemic and semantic distractors are included.

Given food insecurity's linkages with stress, mental health, and impaired executive function [23, 24], the current analysis used the age-standardized composite score for fluid cognition (SEARCH score range 39–146) and the age-standardized sub-domain scores derived from the Picture Vocabulary Test (SEARCH score range 64–151) and the Flanker Inhibitory Control and Attention Test (SEARCH score range 43–134) as main outcomes. Age-standardized Picture Vocabulary Test scores were also included as a covariate in adjusted models given their potential influence and possible bidirectional relationship with fluid cognition [12, 25]. Age-standardized scores have a normative mean of 100 and a standard deviation of 15, and are interpreted as differences in performance across individuals of the same age (https://www.healthmeasures.net/score-and-interpret/interpret-scores/nih-toolbox). The Flanker Inhibitory Control and Attention Test contributes to the composite score for fluid cognition score but scores from the Picture Vocabulary Test do not. The Flanker Inhibitory Control and Attention Test measures one's inhibitory control when asked to focus on a selective visual stimulus. Fluid cognition assesses one's ability to reason, think abstractly, and problem solve in novel situations, independent of previous learning experiences. Crystallized cognition assesses one's application of established knowledge and skills that have been acquired through past education, occupation, or cultural learning experiences over a lifetime, for example, information that is not novel or unique [26]. Trained assessors administered all NIHT-CB tests. In addition, given findings from previous work, the age-standardized score from the Picture Vocabulary Test (receptive language) was used as a surrogate measure of crystallized cognition and included in covariate-adjusted models [12]. Given its validation across a range of cognitive abilities, all SEARCH participants were eligible for NIHT-CB tests [27, 28].

#### 2.2.3. Glycemic Control

Glycemic control was classified based on the American Diabetes Association (ADA) and International Society for Pediatric and Adolescent Diabetes (ISPAD) 2014 Guidelines for HbA1c and included binary categories of higher HbA1c levels (>9% HbA1c) and lower HbA1c levels (≤9% HbA1c) [29]. Whole blood sampling for HbA1c was collected from participants during research visits. Blood samples were analyzed using an automated nonporous ion-exchange high-performance liquid chromatography system (model G-7; Tosoh Bioscience, Montgomeryville, Pennsylvania) [30], with an intra-assay coefficient of variation of 0.047%, an inter-assay coefficient of variation of 0.070%, and a normal reference range of 4.2%–5.8% [20].

#### 2.2.4. Covariates

Race, ethnicity, and sex were obtained primarily through self-report using standard Census questions on the initial participant survey (IPS) or, as a secondary source, from medical record data for those who did not complete the IPS [31, 32]. We considered race and ethnicity as social constructs, and the data were categorized as minority racial and ethnic groups vs. non-Hispanic whites. Diabetes duration was calculated as the difference in months between the date of the current visit and the date of diagnosis, as determined through chart review.

Parent/guardians reported their highest educational degree or level of schooling completed, as well as that of their partner/spouse. Responses were categorized into four categories, including parents having less than a high school graduate versus a high school graduate level of education versus some college/Associate degree versus bachelor's degree or more. Participants (or their parents) were also asked to report their household income. Household income was categorized as <$25,000, $25,000–49,999, $50,000–$74,999, and ≥$75,000. Clinical diabetes type was confirmed based on information obtained from a health care provider and recorded via medical record review.

### 2.3. Statistical Analysis

Data analyses were conducted with SAS 9.4 software (SAS Institute, Cary, NC). Descriptive comparisons between household food insecure and secure were completed by *t*-test, for continuous variables and chi-square for categorical variables, according to diabetes type. Multivariable linear regression was used to assess cross-sectional associations between food insecurity and fluid cognitive function among youth and young adults with T1D or T2D, using models stratified by diabetes type. An unadjusted model was first assessed (Model 1), followed by a model adjusting for race and ethnicity, sex, diabetes duration, and clinic site (Model 2), and models that adjusted for parental education and household income (Model 3) and glycemic control and the Picture Vocabulary Test (Model 4) in addition to Model 2 variables. Finally, a model adjusted for all aforementioned covariates (Model 5). Furthermore, potential effect modification by glycemic control of the food insecurity–fluid cognition association was evaluated, and two interaction models were analyzed. One interaction model adjusted for race and ethnicity, sex, diabetes duration, clinic site, and Picture Vocabulary Test, and the second interaction model additionally adjusted for parental education and household income. All models were stratified by diabetes type. The same multivariable linear regression models were repeated to assess cross-sectional associations between food insecurity and the Picture Vocabulary Test and Flanker Inhibitory Control and Attention Test. However, models were not adjusted for the Picture Vocabulary Test when this measure was considered the outcome.

For Models 2 and 4, the analysis sample included 1,240 youth and young adults with T1D and 334 with T2D who had complete data for measures from the NIHT-CB, household food security status, and covariate variables of glycemic control, race and ethnicity, sex, diabetes duration, and clinic site. After adjustment for household income and parental education (Models 3 and 5), the sample was 1,123 youth and young adults with T1D and 270 with T2D (*Supplementary 1*). Because household income and education had a significant amount of missing responses (income = 27.3%; education = 6.5%) and given that SEARCH is a longitudinal study offering 3–6 data collection points prior to the SEARCH 4 visit for each participant, we substituted the value of household income and education from the most recently available data collection timepoint for the missing timepoints. Sensitivity analyses showed that these two variables were stable over time.

## 3. Results and Discussion

### 3.1. Results

The total sample composition was 57.2% female, 48.9% non-Hispanic white, 34.1% had a household income greater than or equal to $75,000, and 41.9% had a parent with at least a bachelor's degree or higher level of education. The average age was 20.8 (range 10.1–36.0) and 24.6 years (range 11.0–35.6) and the average diabetes duration at the time of SEARCH 4 data collection was 11.2 and 10.3 years, respectively, among youth and young adults with T1D and T2D. Across participants with T1D or T2D, 47.3% had higher HbA1c levels (>9% HbA1c) (Table 1).

Overall, household food insecurity was experienced by 18.3% of participants with T1D and 31.4% of those with T2D. Among youth and young adults with T1D, those reporting food insecurity in the past year differed from the food secure in terms of minority racial and ethnic groups (*p* < 0.0001), clinic site (*p*=0.0388), lower household income (*p* < 0.0001), and lower parental education (*p* < 0.0001). In unadjusted descriptive comparisons, participants with T1D who experienced food insecurity in the last year were also more likely to have a lower average composite fluid cognition score (*p* < 0.05), lower scores on the Picture Vocabulary Test used to assess crystallized cognition (*p*=0.0016), and higher glycemic levels (*p* < 0.0001) than those that were food secure. Among participants with T2D, the only differences between the food secure and the food insecure were the distributions of non-Hispanic white race and ethnicity (*p*=0.0143), clinic site (*p*=0.0281), and lower household income (*p*=0.0008) (Table 2).

On average, composite Fluid Cognition and Picture Vocabulary Test scores were 2.5 and 3.5 points less for food insecure T1D participants compared to those who were food secure, but no differences were observed for Flanker Inhibitory Control and Attention Test scores. Average composite Fluid Cognition, Picture Vocabulary Test, and Flanker Inhibitory Control and Attention Test scores were not significantly different between food insecure versus food secure participants with T2D. In participants with T1D, regression models showed that food insecurity was associated with a lower composite fluid cognition score (*β* = −2.5, standard error (s.e.) = 1.2, 95% confidence interval (CI) = −4.8, −0.1) in the unadjusted model. This association was attenuated to non-significance after adjusting for sex, race and ethnicity, diabetes duration, and clinic site in Model 2 (Table 2). Specifically, race and ethnicity, diabetes duration, and clinic site but not sex accounted for the attenuation in Model 2. Additional adjustments for parental education, household income, glycemic control, and scores on the Picture Vocabulary Test in Models 3 through 5 did not alter the findings. In the final adjusted Model 5, household income and crystalized cognition assessed via the Picture Vocabulary Test were the only significant factors that remained associated with fluid cognition. In these multivariable models, glycemic control was not associated with fluid cognition in participants with T1D (*Supplementary 2*).

Among youth and young adults with T2D, no associations were found between food insecurity and the composite fluid cognition score in unadjusted or adjusted models. Food insecurity was also not associated with the Flanker Inhibitory Control and Attention test score, nor any of the other sub-domains of fluid cognition (i.e., the Dimensional Card Sorting Test, List Sorting Working Memory Test, Picture Sequence Memory Test, and Pattern Comparison Speed Test) in youth and young adults in T1D or T2D. Lastly, food insecurity was not found to be associated with Picture Vocabulary Test score among youth and young adults in T1D or T2D (Table 2). In the final adjusted Model 5, sex, race and ethnicity, clinic site, parental education, household income, and glycemic control remained significantly associated with the Picture Vocabulary Test.

Although not found for other measures of cognition nor among participants with T2D, analyses revealed effect modification by HbA1c in the association between food insecurity and the outcome of the composite fluid cognition score among participants with T1D (*p*=0.0199 for interaction term, Table 3). Focusing first on those with lower HbA1c levels (HbA1c ≤ 9%), participants with T1D who were food insecure had, on average, a lower composite fluid cognition score than those who were food secure (difference = −3.9, 95% CI = −7.3, −0.5) (Table 3) in the model adjusted for sex, race and ethnicity, clinic site, duration of diabetes, glycemic control, and crystallized cognition. However, there was no difference by food security status in the average composite fluid cognition score among those with higher HbA1c levels (HbA1c > 9%) (difference = 1.5, 95% CI = −1.5, 4.5). In the second model, there was additional adjustment for household income and parental education, and this attenuated the interaction (*p*=0.0531). Contrastingly, in a separate analysis where glycemic control was considered as a continuous measure of HbA1c versus binary measure in regression models, effect modification by HbA1c was not found in the association between food insecurity and composite fluid cognition score among participants with T1D for the model adjusted for sex, race and ethnicity, clinic site, duration of diabetes, glycemic control, and crystallized cognition.

### 3.2. Discussion

In this cross-sectional study, food insecurity was inversely related to fluid cognition among youth and young adults with T1D, but this association was largely accounted for by demographic and clinical differences between the food secure and insecure groups, as differences were attenuated once adjusted for race and ethnicity, diabetes duration, and clinic site. After additional adjustment for socioeconomic characteristics, glycemic control, and crystalized cognition assessed via the Picture Vocabulary Test, household income remained associated with fluid cognition. Moreover, receptive language skills (Picture Vocabulary Test), a surrogate measure of crystallized cognition, were an independent predictor of fluid cognition and functioned as a confounder of the relationships between food insecurity and fluid cognition.

Whereas fluid and crystallized cognition both capture components of intellectual functioning, these concepts are distinct. Crystallized cognition is less likely to be perturbed by intervening changes in brain functioning relative to fluid cognition functioning; this has been demonstrated using the NIH-T [33, 34]. Previous research suggests that fluid cognition is linked to stress processes and engagement in lifestyle and health activities [35]. Moreover, household food insecurity is inherently a high-stress state, which motivated this analysis of the association between food insecurity and cognition [12, 23, 36]. Nevertheless, food-insecure youth and young adults with T1D or T2D did not have different fluid or crystallized cognition compared to those who were food-secure after adjustment for confounders. Thus, findings from this cross-sectional research support that household food insecurity does not influence participant's ability to reason and acquire new knowledge nor their ability to apply previous learning experiences. In contrast, a recent study among people without diabetes found that food insecurity was inversely related to general cognitive function after adjusting for age, sex, race/ethnicity, education, marital status, household income, and smoking status; however, a sample of older adults (≥60 years old) was used [24]. In general, few studies have examined similar associations among youth or young adults with or without diabetes and have adjusted for a range of covariates across studies.

In addition to studying fluid cognition and crystallized cognition, several other sub-domains of fluid cognitive function were evaluated in their relationship with household food insecurity, including scores on tests of set shifting (Dimensional Card Sorting Test), inhibitory control (Flanker Inhibitory Control and Attention Test), working memory (List Sorting Working Memory Test), episodic memory (Picture Sequence Memory Test), and speed of processing (Pattern Comparison Speed Test). Differences in these fluid cognitive function domains between youth and young adults with youth-onset T1D and T2D have been described previously [12], and people with diabetes tend to score lower on a variety of fluid cognitive functioning domains compared to persons without diabetes regardless of the measures utilized [37]. A particularly strong case can be made for greater impulsivity and inattention in persons with diabetes versus in those without diabetes [38–42]. Because household food insecurity is also thought to have a substantial impact on not only mental health but also executive function [24], we specifically hypothesized that food insecure people would score more poorly on the Flanker Inhibitory Control and Attention Test, but no differences were observed in our sample in either T1D or T2D.

While household food insecurity was initially associated with both fluid and crystallized cognition, in the final adjusted models, the association was ultimately explained by socioeconomic characteristics, in particular household income. Parental education and demographic characteristics (race and ethnicity) were also significantly associated with crystallized cognition in the final adjusted models. While some attenuation is expected given the framing of food insecurity as an economic issue in the assessment, some residual or independent effects of food insecurity on a health outcome among youth and young adults with diabetes were anticipated given previous findings of marked, independent associations of food insecurity with glycemic control and diabetes ketoacidosis [18, 43]. Some of these independent effects of food insecurity may be working through lack of access to educational resources or due to vulnerability to other stressors related to poverty that may have led to impaired maturation of brain structures, which are key to performance on tests for cognition [44–46]. In totality, however, these findings suggest that upstream, socioeconomic influences are likely playing an overwhelming role in their impact on cognition over time [44, 46, 47]. Notably, in this study, food insecurity was recalled over the last 12 months. Thus, this relatively short time period for measuring food security status may not be adequate to observe changes in cognition that may be linked to food insecurity.

An additional complicating factor in persons with diabetes is that poor diabetes self-management manifesting in higher HbA1c levels has been shown to have a detrimental impact on cognition [14, 48]. Moreover, the relationships between food insecurity, glycemic control, and cognition in people with diabetes are likely complex. In a recent systematic review, He et al. [14] provided evidence for a relationship between glycemic control and overall cognition and memory among youth with T1D. Specifically, compared to youth with T1D without hypoglycemic episodes, those with severe hypoglycemic episodes had increased overall cognitive deficits (*p*=0.020) and lower performance in memory (*p*=0.032) [14]. Thus, while glycemic control could function as a moderator in the relationship between food insecurity and cognition, food insecurity could have a direct effect on cognition, particularly on executive function and impulse control, especially if recurrent or prolonged [49]. Our earlier SEARCH report by Shapiro et al. [12] found no association between HbA1c and fluid cognition nor an association of hypoglycemic events and fluid cognition among SEARCH 4 youth and young adults with diabetes when diabetes type was included in the multivariable model. Moreover, in contrast to findings from studies conducted among populations without diabetes which support an association between food insecurity and various measures of cognitive function [50, 51], the current study found no relationship between food insecurity and fluid cognitive function among youth and young adults with T1D or T2D regardless of glycemic control. In our study, given that no relationship was found between food insecurity and fluid cognitive function and no association was found between glycemic control and cognitive function [12], moderation of food insecurity's impact on cognition through glycemic control was not supported by our data.

This study has several limitations, including not having measurements of glycemic control based on daily self-monitoring, not having a control group of youth and young adults without diabetes for analyses, and having a cross-sectional design that does not allow for assessment of temporal relationships between food insecurity and cognition outcomes. The measures of cognition were briefer than those traditionally used in the literature. In addition, this research did not examine the association of food insecurity, glycemic control, and cognitive function longitudinally. Thus, it is possible that the magnitude and significance of current study results may change if observed over time and during different neurodevelopmental periods. Among the strengths of this research is the fact that this study used a large sample of youth and young adults with T1D and is one of the largest studies of youth-onset T2D. Validated scales were also used to measure food insecurity and cognitive function.

## 4. Conclusions

As one of the first studies to examine such associations, we found that composite fluid cognition, and sub-domain scores for Receptive Language and Inhibitory Control and Attention did not differ among food insecure versus food secure youth and young adults with T1D or T2D after adjusting for covariates. Findings suggest that socioeconomic characteristics explain associations between household food insecurity and cognition among youth and young adults with T1D; however, further research is needed to better understand these associations over time and among those with T2D. Nevertheless, these findings have potential translational significance, as food insecurity can be ameliorated by access to increased financial resources, with federal food assistance programs such as the Supplemental Nutrition Assistance Program and nonprofit programs and interventions that have food pantries or food vouchers for youth and young adults or families with children being just a few examples [52–54]. In addition to food insecurity, findings suggest a need to identify and address other health-related social needs and sociodemographic factors in youth and young adults with diabetes within clinical practice. Given the rise in food insecurity within U.S. households related to the recent effects of the coronavirus pandemic, future research on food insecurity and links to cognition is especially warranted and should potentially explore differences related to glycemic control status.

## Figures and Tables

**Table 1:** Sample characteristics of youth and young adults with youth-onset type 1 or type 2 diabetes in the SEARCH study, according to household food insecurity.

Characteristic	Overall	Type 1 diabetes		*p* Value	Type 2 diabetes		*p* Value
		Food insecure	Food secure		Food insecure	Food secure	
	*n* = 1,574	*n* = 227	*n* = 1,013		*n* = 104	*n* = 230	
		18.3%	81.7%		31.4%	68.6%	
Age (years), mean	20.8	21.0	20.8	0.6174	25	24.5	0.2812
Sex (%)				0.3497			0.2633
Female	57.2	56.8	53.4		73.1	67.0	
Male	42.8	43.2	46.6		26.9	33.0	
Race and ethnicity (%)				<0.0001 ^*∗*^			0.0143 ^*∗*^
Minority racial and ethnic groups^a^	51.5	55.5	40.7		74.0	85.2	
Non-Hispanic White	48.9	44.5	59.3		26.0	14.8	
Glycemic control (%)				<0.0001 ^*∗*^			0.6792
Higher HbA1c levels	47.3	58.8	41.8		57.7	55.3	
Lower HbA1c levels	52.7	41.2	58.2		42.3	44.7	
Diabetes duration (months), mean	133.9	134.1	133.8	0.9230	122.6	124.5	0.7178
Take any insulin (%)				0.0358 ^*∗*^			0.7807
No	10.1	2.2	0.7		43.1	44.8	
Yes	89.9	97.8	99.3		56.9	55.2	
Clinic site (%)				0.0388 ^*∗*^			0.0281 ^*∗*^
1	23.1	25.1	18.0		37.5	37.0	
2	16.3	13.7	16.6		16.4	17.8	
3	30.3	30.4	33.1		25.0	20.4	
4	17.8	13.7	18.4		11.5	21.7	
5	12.5	17.2	13.9		9.6	3.0	
Household income^b^ (%)				<0.0001 ^*∗*^			0.0008 ^*∗*^
<$25,000	25.9	35.9	16.5		58.5	46.1	
$25,000–$49,000	24.9	34.9	20.0		38.3	32.0	
$50,000–$74,000	15.1	20.7	16.1		2.1	9.5	
$75,000+	34.1	8.5	47.4		1.1	12.4	
Education^b,c^ (%)				<0.0001 ^*∗*^			0.8842
<HS	7.3	7.1	5.8		11.5	12.2	
HS graduate	18.4	21.1	12.7		32.7	34.9	
Some college	32.4	40.5	28.9		40.4	35.8	
Bachelor's degree	41.9	31.3	52.6		15.4	17.0	
HbA1c (%), mean	9.2	9.9	9.0	<.0001 ^*∗*^	9.3	9.6	0.4034
Composite fluid cognition, mean	93.2	93.7	96.2	0.0406 ^*∗*^	84.3	83.4	0.6688
Picture vocabulary (crystallized), mean	101.0	100.9	104.4	0.0018 ^*∗*^	92.5	90.1	0.1448
Flanker inhibitory control and attention (fluid), mean	83.6	84.1	85.3	0.2186	79.0	77.6	0.3591

^a^Minority race includes Hispanic and non-Hispanic minority race and ethnic groups. ^b^Total will not equal 1,574 for this variable due to missing data. ^c^Highest Education level of parent.  ^*∗*^Indicates statistical significance of *p* < 0.05.

**Table 2:** Association between household food security status and measures of cognitive function among youth and young adults with type 1 or type 2 diabetes (T2D) in the SEARCH study.

Adjustments	Type 1 diabetes (*n* = 1,240)			Type 2 diabetes (*n* = 334)		
	Composite fluid cognition	Flanker inhibitory control and attention	Picture vocabulary (crystallized) ^*∗*^	Composite fluid cognition	Flanker inhibitory control and attention	Picture vocabulary (crystallized) ^*∗*^
Model 1						
*β*^1 a^	−2.5	−1.1	−3.4	0.9	1.4	2.4
Se	1.2	0.9	1.1	2.1	1.6	1.6
*p* Value	0.0406 ^*∗*^	0.2186	0.0018 ^*∗*^	0.6688	0.3591	0.1448
Model 2						
Adj *β*^2 b^	−1.4	−0.7	−1.7	−0.4	0.6	1.6
Se	1.2	0.9	1.0	2.1	1.6	1.6
*p* Value	0.2435	0.4323	0.1025	0.8378	0.6920	0.2872
Model 3 ^*∗*^ ^*∗*^						
Adj *β*³ ^c^	1.2	0.8	0.8	0.5	−0.1	3.2
Se	1.3	1.0	1.1	2.3	1.8	1.7
*p* Value	0.3277	0.4212	0.4527	0.8168	0.9501	0.6093
Model 4 ^*∗*^ ^*∗*^ ^*∗*^						
Adj *β*^4 d^	−0.9	−0.5	−0.9	−1.9	0.3	1.6
Se	1.1	0.9	1.0	1.8	1.5	1.6
*p* Value	0.4324	0.5878	0.3891	0.3084	0.8640	0.3071
Model 5 ^*∗*^ ^*∗*^^,^ ^*∗*^ ^*∗*^ ^*∗*^						
Adj *β*^5 e^	0.9	0.6	1.1	−2.0	−1.7	3.2
Se	1.2	1.0	1.1	2.1	1.7	1.7
* p* Value	0.4614	0.5321	0.3022	0.3373	0.3213	0.0649

^a^No adjustment, crude model. ^b^Model 2 controlled for sex, race and ethnicity, clinic site, duration of diabetes. ^c^Model 3 controlled for sex, race and ethnicity, clinic site, duration of diabetes, parent education and household income. ^d^Model 4 controlled for sex, race and ethnicity, clinic site, duration of diabetes, glycemic control, crystallized cognition. ^e^Model 5 controlled for sex, race and ethnicity, clinic site, duration of diabetes, parent education, household income, glycemic control, and crystallized cognition.  ^*∗*^Indicates statistical significance of *p* < 0.05.  ^*∗*^ ^*∗*^Due to missing values for household income and parental education, Model 3 and 5 included a sample of 1,123 participants with type 1 diabetes and 270 of those with type 2 diabetes.  ^*∗*^ ^*∗*^ ^*∗*^ Models 4 and 5 did not control for crystalized cognition when the outcome of interest was crystallized cognition.

**Table 3:** Difference in average composite fluid cognition scores among youth and young adults with type 1 diabetes for models which included the interaction of household food security status and glycemic control.

Glycemic control (higher vs. lower HbA1c levels) and household food security status (food insecure vs food secure) interaction model	Estimate (95% CI)	*p* Value
Model 6^a^ (*n* = 1,240)		0.0199 ^*∗*^
Higher HbA1c levels for food insecure vs. food secure	1.46 (−1.54, 4.46)	0.3394
Lower HbA1c levels for food insecure vs. food secure	−3.89 (−7.26, −0.52)	0.0239 ^*∗*^
Model 7^b,c^ (*n* = 1,123)		0.0531
Higher HbA1c levels for food insecure vs. food secure	2.95 (−0.24, 6.14)	0.0702
Lower HbA1c levels for food insecure vs. food secure	−1.70 (−5.30, 1.90)	0.3548

^a^Model 6 controlled for sex, race and ethnicity, clinic site, duration of diabetes, glycemic control, crystallized cognition and included the interaction of food insecurity and glycemic control. ^b^Model 7 controlled for sex, race and ethnicity, clinic site, duration of diabetes, glycemic control, crystallized cognition, parental education, household income, and included the interaction of food insecurity and glycemic control. ^c^Due to missing values for household income and parental education, Model 7 included a sample of 1,123 participants with type 1 diabetes.  ^*∗*^Indicates statistical significance of *p* < 0.05.

## Data Availability

Data described in this manuscript, code book, and analytic code will be made available upon publication to investigators whose proposed use of the data has been approved by an independent review committee. Proposals should be directed to the corresponding author, Angela D. Liese, at liese@sc.edu. To gain access, data requesters will need to sign a data use agreement.

## Supplementary Materials

Supplementary 1 Completed STROBE checklist for cross-sectional studies.

Supplementary 2 Main effects Model 2 and Model 5 of predictors of Fluid Cognition Score among SEARCH 4 youth and young adults with type 1 diabetes.
