# Supplementary material for: Household Food Insecurity and Cognition in Youth and Young Adults with Youth-Onset Diabetes
Source: Pediatr Diabetes. 2023 Sep 14;2023:6382663. doi: 10.1155/2023/6382663 (PMC11100256; doi:10.1155/2023/6382663)
Supplement: Supplementary 1 — Completed STROBE checklist for cross-sectional studies. [file 6382663.f1.doc]

STROBE Statement—Checklist of items that should be included in reports of ***cross-sectional studies***

|  | Item No | Recommendation |
| --- | --- | --- |
| **Title and abstract** | 1 | (*a*) Indicate the study’s design with a commonly used term in the title or the abstract – Study design has been mentioned in abstract in page 3, lines 4-6. |
|  |  | (*b*) Provide in the abstract an informative and balanced summary of what was done and what was found – Addressed on page 3, lines 1-22 |
| Introduction | | |
| Background/rationale | 2 | Explain the scientific background and rationale for the investigation being reported – Addressed on page 4 & 5, lines 28-60 |
| Objectives | 3 | State specific objectives, including any prespecified hypotheses –addressed on page 5, lines 61-69 |
| Methods | | |
| Study design | 4 | Present key elements of study design early in the paper – addressed in study subjects subsection on page 5&6, lines 73- 87 |
| Setting | 5 | Describe the setting, locations, and relevant dates, including periods of recruitment, exposure, follow-up, and data collection – discussed in study subjects subsection in page 5 & 6, lines 73-97 |
| Participants | 6 | (*a*) Give the eligibility criteria, and the sources and methods of selection of participants – addressed in study subjects subsection on page 5&6 lines 73- 87 |
| Variables | 7 | Clearly define all outcomes, exposures, predictors, potential confounders, and effect modifiers. Give diagnostic criteria, if applicable – discussed in study covariates subsection on page 8 lines 144-157 |
| Data sources/ measurement | 8* | For each variable of interest, give sources of data and details of methods of assessment (measurement). Describe comparability of assessment methods if there is more than one group - discussed in study measures subsection on pages 6&7 lines 99-143 |
| Bias | 9 | Describe any efforts to address potential sources of bias – addressed in sample selection in lines 73 – 85 and analysis, lines 186-187 |
| Study size | 10 | Explain how the study size was arrived at – discussed in study subjects subsection in page 6, lines 93-97 |
| Quantitative variables | 11 | Explain how quantitative variables were handled in the analyses. If applicable, describe which groupings were chosen and why - discussed in analysis section on pages 8&9 lines 159-187 |
| Statistical methods | 12 | (*a*) Describe all statistical methods, including those used to control for confounding – in lines 159-176 and 186-187 |
|  |  | (*b*) Describe any methods used to examine subgroups and interactions – in lines 159-176 |
|  |  | (*c*) Explain how missing data were addressed – in line 177 – 187 |
|  |  | (*d*) If applicable, describe analytical methods taking account of sampling strategy – in line 177-187 |
|  |  | (*e*) Describe any sensitivity analyses – in line 186-187 |
| Results | | |
| Participants | 13* | (a) Report numbers of individuals at each stage of study—eg numbers potentially eligible, examined for eligibility, confirmed eligible, included in the study, completing follow-up, and analysed – in lines 73-97 |
|  |  | (b) Give reasons for non-participation at each stage – in lines 73-97 |
|  |  | (c) Consider use of a flow diagram – not used |
| Descriptive data | 14* | (a) Give characteristics of study participants (eg demographic, clinical, social) and information on exposures and potential confounders – in lines 191-197 |
|  |  | (b) Indicate number of participants with missing data for each variable of interest – in line 177 – 187 |
| Outcome data | 15* | Report numbers of outcome events or summary measures – in lines 210-221 |
| Main results | 16 | (*a*) Give unadjusted estimates and, if applicable, confounder-adjusted estimates and their precision (eg, 95% confidence interval). Make clear which confounders were adjusted for and why they were included – in lines 191-209 |
|  |  | (*b*) Report category boundaries when continuous variables were categorized – in lines 191-209 |
|  |  | (*c*) If relevant, consider translating estimates of relative risk into absolute risk for a meaningful time period – not used |
| Other analyses | 17 | Report other analyses done—eg analyses of subgroups and interactions, and sensitivity analyses – in lines 222-252 |
| Discussion | | |
| Key results | 18 | Summarise key results with reference to study objectives – in lines 254-262 |
| Limitations | 19 | Discuss limitations of the study, taking into account sources of potential bias or imprecision. Discuss both direction and magnitude of any potential bias – in lines 326-336 |
| Interpretation | 20 | Give a cautious overall interpretation of results considering objectives, limitations, multiplicity of analyses, results from similar studies, and other relevant evidence – in lines 263-325 |
| Generalisability | 21 | Discuss the generalisability (external validity) of the study results – in lines 339 - 349 |
| Other information | | |
| Funding | 22 | Give the source of funding and the role of the funders for the present study and, if applicable, for the original study on which the present article is based – addressed in funding statement section page 14, lines 384-392 |

*Give information separately for exposed and unexposed groups.

**Note:** An Explanation and Elaboration article discusses each checklist item and gives methodological background and published examples of transparent reporting. The STROBE checklist is best used in conjunction with this article (freely available on the Web sites of PLoS Medicine at http://www.plosmedicine.org/, Annals of Internal Medicine at http://www.annals.org/, and Epidemiology at http://www.epidem.com/). Information on the STROBE Initiative is available at www.strobe-statement.org.
