# Supplementary material for: Household Food Insecurity and Cognition in Youth and Young Adults with Youth-Onset Diabetes
Source: Pediatr Diabetes. 2023 Sep 14;2023:6382663. doi: 10.1155/2023/6382663 (PMC11100256; doi:10.1155/2023/6382663)
Supplement: Supplementary 2 — Main effects Model 2 and Model 5 of predictors of Fluid Cognition Score among SEARCH 4 youth and young adults with type 1 diabetes. [file 6382663.f2.docx]

**Supplement 2.** Main effects Model 2 and Model 5 of predictors of Fluid Cognition Score among SEARCH 4 youth and young adults with type 1 diabetes

| N=1240 | Model 2 | | | Model 5 | | |
| --- | --- | --- | --- | --- | --- | --- |
| Characteristic | β (SE) | t | p-value | β (SE) | t | p-value |
| Intercept | 103.59 (1.81) | 57.30 | **<0.0001** | 60.29 (4.47) | 13.48 | **<0.0001** |
| Food Insecure | -1.39 (1.19) | -1.17 | 0.2435 | 0.92 (1.25) | 0.74 | 0.4614 |
| Food Secure | ref | - | - | ref | - | - |
| Female sex | 0.39 (0.92) | 0.42 | 0.6738 | 1.45 (0.93) | 1.56 | 0.1197 |
| Male sex | ref | - | - | ref | - | - |
| Minority race/ethnicity | -6.65 (0.97) | -6.85 | **<0.0001** | -1.76 (1.08) | -1.63 | 0.1043 |
| Non-Hispanic White race/ethnicity | ref | - | - | ref | - | - |
| Clinic Site 1 | -4.71 (1.31) | -3.59 | **0.0003** | -3.19 (1.33) | -2.40 | **0.0168** |
| Clinic Site 2 | -3.21 (1.39) | -2.30 | **0.0214** | -2.15 (1.40) | -1.54 | 0.1246 |
| Clinic Site 4 | -1.59 (1.37) | -1.16 | 0.2469 | -1.53 (1.40) | -1.09 | 0.2739 |
| Clinic Site 5 | 1.82 (1.43) | 1.27 | 0.2054 | -0.62 (1.46) | -0.43 | 0.6681 |
| Clinic Site 3 | ref | - | - | ref | - | - |
| Diabetes Duration | -0.03 (0.01) | -2.29 | **0.0222** | -0.01 (0.01) | -1.25 | 0.2134 |
|  |  |  |  |  |  |  |
| Bachelor’s degree parental education |  |  |  | 1.73 (1.12) | 1.55 | 0.1216 |
| HS graduate parental education |  |  |  | -0.18 (1.48) | -0.12 | 0.9038 |
| < HS graduate parental education |  |  |  | -2.32 (2.19) | -1.06 | 0.2910 |
| Some college parental education |  |  |  | ref | - | - |
| $25,000-$49,000 |  |  |  | 2.37 (1.42) | 1.67 | 0.0950 |
| $50,000-$74,000 |  |  |  | 2.87 (1.57) | 1.82 | 0.0688 |
| $75,000+ |  |  |  | 4.95 (1.46) | 3.38 | **0.0007** |
| < $25,000 |  |  |  | ref | - | - |
| Higher HbA1c Levels |  |  |  | 1.17 (0.97) | 1.20 | 0.2295 |
| Lower HbA1c Levels |  |  |  | ref | - | - |
| Picture Vocabulary (crystallized) |  |  |  | 0.33 (0.04) | 9.40 | **<0.0001** |
